# Recommendations and Testimonials

**Published:** 1908-06-20

**Authors:** 


### Nursing Administration.

### RECOMMENDATIONS AND TESTIMONIALS.

The duties of the matron in regard to the perpetual flow of the staff through the institution she superintends are serious. She is ceaselessly called upon to sum up the character and capabilities of the people who have been working under her, and is often expected not merely to pronounce on the work and abilities of the person in question, but to state what opinion she has formed of the work likely to be accomplished by her under quite different conditions. The matron has, on the one hand, to discharge her duty to the woman who has been filling a post passably, if not perfectly, under her superintendence, and who relies upon her good word as a principal stock-in-trade in seeking new work. She has, on the other hand, to be mindful of her duty to the new employer, who depends often with embarrassing confidence on the sincerity of the applicant's former matron. Many an anxious moment is spent in considering the pros and cons of doubtfully efficient nurses by conscientious matrons, and there are few indeed who can profess themselves entirely satisfied with the reports they have in all cases supplied. It would simplify matters very much if people would clearly understand that no one ought to be expected to make forecasts, under cover of granting testimonials to members of their staff. The very wisest matrons go astray when it comes to prophesying about their subordinates. Every one can quote instances of adverse opinions delivered against nurses, who have subsequently risen to the highest distinction in their profession. It is only human nature that those in command should be disposed to prophesy success for the workers who melt, as it were, into their personality, who make efficient echoes of their opinions and efficient transmitters of orders And yet it is not always these who respond most readily to the call of responsibility. The applicant has perhaps shown genuine love and zeal under the matron who is called on to recommend her. What more natural than to take it for granted, often with disastrous consequences, that the same affectionate energy will be at the service of the next institution she serves? Again, the silent and apparently cold nurse with a reserve force of energy which has as yet found no special outlet, often succeeds, contrary to all expectation, in a position of authority. And in general, it may be maintained that there is nothing more precarious than attempts to foretell how people will deal with circumstances, and nothing more difficult than to understand the characters of fellow-workers. Truth to tell, impartiality is exceedingly difficult in the case of persons who have lived together in the close intimacy of institution life. There is such a thing as knowing people's little faults too well, and when it comes to deciding as to the future capabilities, the jarring contact with such little faults blinds the eyes to other factors in the character. For this reason no one would rely on the opinion of a woman's own family about her fitness for a given post.

The difficulties of the matron when called upon to recommend applicants for particular kinds of work, are likely to be seriously multiplied if the proposed scheme for forming a Territorial Nursing Reserve, and enlarging the Army Nursing Reserve, is based on the plan of calling for volunteers from hospitals and other institutions, who shall be pledged to come out when called on. When certain regulations respecting age, health, and training have been satisfied, it is by the matron's report that the governing committee will be guided in placing the name of a nurse on the roll. Matrons will therefore be called on to make a series of confidential reports to the authorities on the suitability of all their nurses who may be fired with the novel idea of going to the wars, and they will be urged to express an opinion, not only on the legitimate subject of how these nurses have done and are doing the work entrusted to them, but how far they are suited to undertake work which is quite outside their present range of experience. What is still more unreasonable, the matron will be called on to forecast, not merely how the applicant is at present qualified to succeed in this untried work, but how she is likely to succeed with it in the course of an unknown term of years, when, if ever, she is called up for service. Under such conditions it is apparent that the recommendations granted will be merely waste paper; but that they will cost the conscientious considerable discomfort is inevitable. Let it be supposed that a probationer has passed through her course with credit, yet the matron perceives she has not the balance (to sum up an indefinable series of qualities) essential in a nurse called upon to nurse soldiers in time of war. The nurse in question will deem it a shameful injustice if the matron declines to recommend her for the Reserve, and it is not clear that the nurse may not be right. The individual in question may be a woman who develops late, and only needs to have responsibility thrown upon her to turn into a very fine character. In five years' time she may be very suitable for Army nursing purposes, and her preferred comrade may have deteriorated to an equal extent. The onus of recommending people for posts of an unknown nature in the future, ought not to be thrown on the heads of training schools, and in our opinion all recommendations ought to be confined with extreme care to an enumeration of the qualities which have come under actual observation in the institution in question, leaving guesses, whether favourable or otherwise about the future, severely alone. For this reason we deprecate in so delicate a matter as the choice of nurses in any future war, the attempt to forecast the fitness of a large body of women for work which needs all the best qualities of nurses who are in the very height of their powers. We are convinced that matrons will incur the responsibility of making such recommendations with the greatest reluctance, and we think this reluctance should be respected.

